# P-951. Cefazolin Monotherapy versus Standard of Care in Type III Open Fractures

**DOI:** 10.1093/ofid/ofaf695.1153

**Published:** 2026-01-11

**Authors:** Tyler Ackley, Casey J Dempsey, Matthew Morrison

**Affiliations:** Hartford Hospital, Hartford, CT; Hartford Healthcare, Hartford, Connecticut; Hartford Hospital, Hartford, CT

## Abstract

**Background:**

Gustilo-Anderson type III open fractures correspond to the most severe fracture injuries – characterized by extensive soft tissue damage or those complicated by vascular injury or severe contamination. While cefazolin monotherapy remains the mainstay of therapy for type I and II open fractures, the Eastern Association for the Surgery of Trauma (EAST) guideline recommends broadening therapy in type III open fractures, traditionally with an aminoglycoside. Alternatively, however, the Surgical Infection Society (SIS) states that there is a lack of sufficient data to routinely target Gram-negative bacteria in this setting. The objective of this study was to compare key outcomes for patients with type III open fractures, such as infection rates and acute kidney injury (AKI) in patients treated with an aminoglycoside versus those that received an aminoglycoside-sparing regimen.

**Methods:**

This was a multi-center chart review of patients admitted with a primary diagnosis of type III open fracture identified via ICD-10 code. Patients were excluded if they presented to an outside hospital emergency department or had been on antibiotics prior to their admission. Chart review was completed on patients admitted from August 20th, 2016 to May 1st, 2023. The primary endpoint of the study was 30-day injury site infections. Secondary endpoints, including the development of AKI and the rate of Gram-negative infections were also assessed.

**Results:**

217 patients were included for analysis, of which 183 received aminoglycoside therapy. Baseline characteristics including patient comorbidities, rate of intensive care unit admission, and receipt of vasopressors were well balanced between groups. There was a non-statistically significant increase in infections in the patients receiving aminoglycosides (18.0% vs 8.8%, p=0.283). Similarly, the rate of AKI was also numerically higher in the aminoglycoside-treated groups (7.1% vs 2.9%, p=0.598).

**Conclusion:**

Cefazolin monotherapy had similar rates of surgical site infections when compared to cefazolin plus an aminoglycoside. Rates of AKI were numerically lower in the cefazolin monotherapy arm. Cefazolin monotherapy may represent a reasonable alternative to aminoglycoside combination therapy in patients with type III open fractures.

**Disclosures:**

All Authors: No reported disclosures

